# Myocardial hypertrophy: the differentiation of uremic, hypertensive, and hypertrophic cardiomyopathies by cardiac MRI

**DOI:** 10.1186/s13244-024-01770-0

**Published:** 2024-08-01

**Authors:** Zhaoxin Tian, Shiqi Jin, Huaibi Huo, Yue Zheng, Yue Li, Hui Liu, Zhaodi Geng, Shutong Liu, Shinuo Li, Zequn Liu, Xinru Wang, Ting Liu

**Affiliations:** Department of Radiology, The First Hospital of China Medical University, Shenyang, China

**Keywords:** Myocardium, Cardiomyopathies, Magnetic resonance imaging, Global longitudinal strain

## Abstract

**Objectives:**

To apply cardiac magnetic resonance imaging (CMR) for detailed myocardial characterization in uremic cardiomyopathy (UC), hypertensive cardiomyopathy (HTN), and hypertrophic cardiomyopathy (HCM) aiming to enrich the understanding of UC’s etiology and further support the development of therapeutic strategies.

**Methods:**

A total of 152 patients (age: 49.2 ± 9.9 years; 65.8% male) underwent routine CMR from June 2016 to March 2023. Retrospectively, 53 patients with UC, 39 patients with HTN, 30 patients with HCM, and 30 healthy controls were included. Functional analysis, feature tracking of the left ventricle and left atrium, and myocardial T1, T2, and T2* mapping were performed. Statistical analysis included Pearson correlation and ROC analysis to define correlations and discriminators between groups.

**Results:**

UC patients demonstrated significantly higher native T1 (*p* < 0.001 for all) and T2 (*p* < 0.002 for all) values compared with the other three groups. UC patients revealed higher left atrial reservoir strain rate (*p* < 0.001 for all) and left atrial conduit strain rate (*p* < 0.001 for all) absolute values as compared with HTN and HCM patients. A significant correlation between T1 and T2 values in UC patients (*r* = 0.511, *p* < 0.001) was found. The combination of T1 values and strain parameters was the best discriminator between UC and HTN patients (AUC = 0.872, 95% CI: 0.801–0.943) and between UC and HCM patients (AUC = 0.840, 95% CI: 0.746–0.934).

**Conclusion:**

UC reveals distinguishing tissue characteristics as evidenced by T1 and T2 mapping, as well as distinguishing functional strain parameters as compared with other hypertrophic phenotypes such as HTN and HCM.

**Critical relevance statement:**

The use of CMR imaging in UC patients offers incremental information to elucidate its complex etiology, contributing to ongoing discourse on effective treatment pathways.

**Key Points:**

- This study investigated uremic, hypertensive, and hypertrophic cardiomyopathies using cardiac MRI.
- UC patients have higher T1 and T2 values and better preserved cardiac function.
- Combined strain and T1 values distinguish UC from other cardiomyopathies.

**Graphical Abstract:**

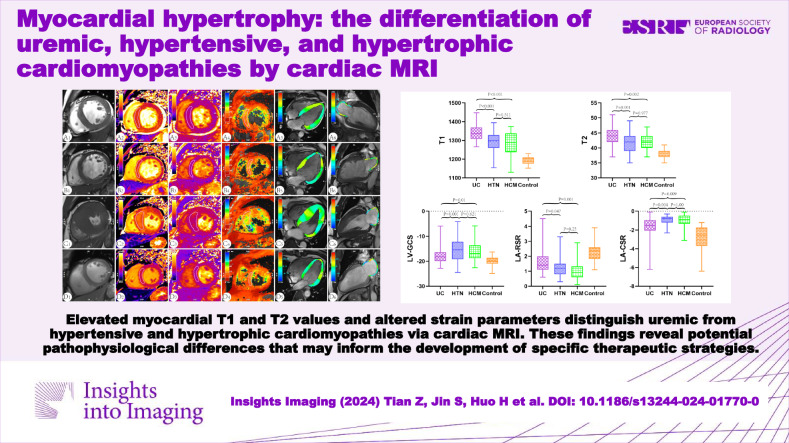

## Introduction

Cardiac disease is a frequent cause of death in patients with end-stage renal disease (ESRD) [1–3]. Uremic cardiomyopathy (UC) manifests itself mainly as left ventricular (LV) hypertrophy, diastolic dysfunction, and heart failure [4, 5]. It involves a complex pathophysiological mechanism, and the primary etiological factors remain unknown at present. Investigating the primary pathogenic factors is of substantial significance for the treatment and rehabilitation of it. The advancement of cardiac magnetic resonance imaging (CMR) enables us to obtain more myocardial histological characteristics non-invasively, facilitating further etiological analysis.

CMR is well suited to assess myocardial tissue characteristics by T1, T2, and T2* mapping, as well as functional parameters of the left ventricle and left atrium by analyzing global function and strain analysis in a wide range of cardiomyopathies. Parametric analysis of native T1 values can detect myocardial diffuse changes and reveal early myocardial fibrosis, edema, and infiltrative disease [6]. Native T2 values are mainly used to evaluate myocardial edema and inflammatory changes [7]. In addition, T2* mapping can detect and quantify iron deposition at an early stage [8, 9]. CMR is also well suited to assess global and regional functional parameters of the left ventricle and left atrium. In particular, CMR feature tracking (CMR-FT) [10, 11] can obtain myocardial strain and strain rate which will further characterize global LV and left atrial (LA) function and may detect early changes in cardiac function [12, 13].

The etiology of UC may be complicated by co-existing hypertension, persistent volume overload, fluctuating volume status, and uremic toxin accumulation [14, 15]. An increase in wall stress-driven hypertensive cardiomyopathy (HTN) and genetically determined hypertrophic cardiomyopathy (HCM) manifest themselves also as hypertrophic phenotypes. Distinguishing various hypertrophic phenotypes is a clinical challenge. CMR is the preferred imaging method to characterize various forms of hypertrophic phenotype and may be helpful in diagnosing specific cardiomyopathies by parametric imaging and functional analysis. We hypothesized that UC may be characterized by specific CMR characteristics assessed with myocardial tissue mapping and functional strain analysis in distinction to hypertensive and genetically determined HCM.

Accordingly, we investigated the unique myocardial tissue characteristics and functional attributes in UC patients through CMR, aiming to discern etiology-specific differences when compared to the myocardial profiles seen in hypertensive and HCM.

## Material and methods

### Study population

This observational and retrospective study included 53 patients with UC, 30 patients with HTN, 39 patients with HCM, and 30 healthy controls who underwent a clinically indicated CMR scan at The First Hospital of China Medical University between June 2016 and March 2023 (Table 1).

**Table 1:** Baseline clinical characteristics

Variables	UC, (*n* = 53)	HTN, (*n* = 39)	HCM, (*n* = 30)	Control, (*n* = 30)	*p*
age (years)	46.40 ± 8.65	51.90 ± 11.00	51.30 ± 9.41	48.33 ± 9.99	0.070
Male, *n* (%)	40 (78.43%)	22 (57.89%)	18 (60.00%)	19 (63.33%)	0.260
BMI (kg/m^2^)	25.41 ± 4.67	25.48 ± 3.89	24.57 ± 3.45	25.36 ± 3.34	0.096
hsTnI (pg/mL)	0.02 ± 0.04	0.03 ± 0.07	0.04 ± 0.05	–	0.129
BNP (pg/mL)	148.00 (84.00–287.00)^b^	101.00 (43.00–187.00)^b^	537.50 (245.50–870.50)	–	< 0.001
eGFR	4.40 ± 1.85^a,b^	96.70 ± 8.30	96.15 ± 7.42	–	< 0.001
Hypertension, *n* (%)	51 (96.20%)^b,c^	39 (100%)^c^	14 (46.70%)^c^	0	0.026
Dyslipidemia, *n* (%)	38 (71.7%)	20 (51.3%)	17 (56.7%)	0	0.062
Diabetes Mellitus, *n* (%)	26 (49.1%)	15 (38.5%)	5 (16.7%)	0	0.069
Smokers, *n* (%)	30 (56.6%)	16 (41%)	11 (36.7%)	13 (43.3%)	0.273
Systolic BP	146.62 ± 12.81^b,c^	140.59 ± 11.19^b,c^	128.67 ± 8.65^c^	111.5 ± 10.02	< 0.001
Diastolic BP	95.28 ± 10.90^a,b,c^	90.15 ± 5.29^b,c^	75.77 ± 6.80	75.13 ± 7.24	0.023

Data are reported as mean ± SD, median (IQR), or *n* (%) as appropriate

*UC* uremic cardiomyopathy, *HTN* hypertension, *HCM* hypertrophic cardiomyopathy, *BMI* body mass index, *hsTnI* high-sensitivity cardiac troponin I, *BNP* brain natriuretic peptide, *eGFR* estimated glomerular filtration rate, *Systolic BP* systolic blood pressure, *Diastolic BP* diastolic blood pressure

^a^ *p* < 0.05 vs HTN

^b^ *p* < 0.05 vs HCM

^c^ *p* < 0.05 vs control

**Fig. 1:**
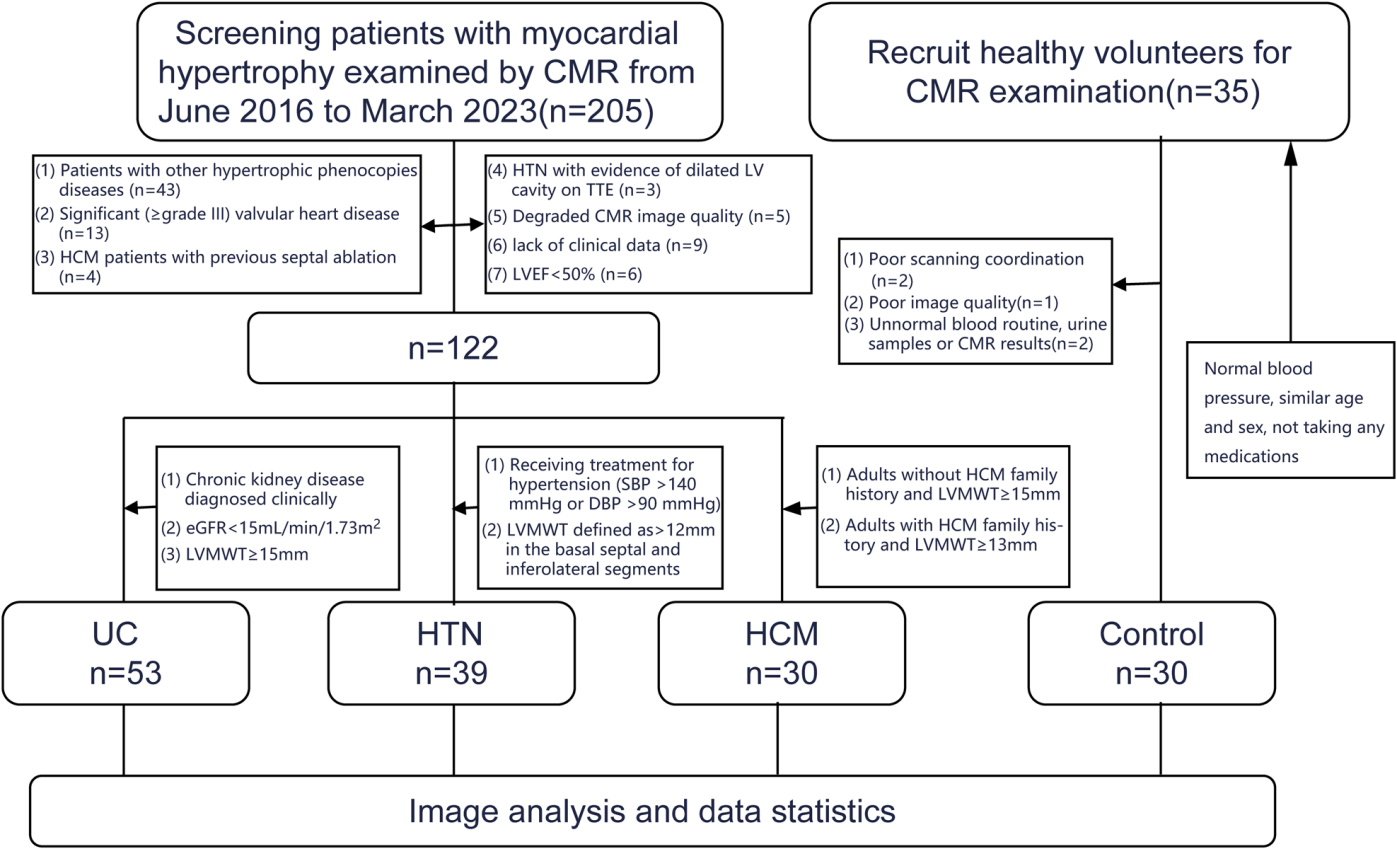
Patient flowchart. The flowchart shows the involvement of patients and controls. UC, uremic cardiomyopathy; HTN, hypertension; HCM, hypertrophic cardiomyopathy; CMR, cardiac magnetic resonance; TTE, transthoracic echocardiography; LVEF, left ventricular ejection fraction; eGFR, estimated glomerular filtration rate; LVMWT, left ventricular maximal wall thickness; SBP, systolic blood pressure; DBP, diastolic blood pressure

The UC patients were defined as suffering from ESRD (estimated Glomerular filtration rate < 15 mL/min/1.73 m^2^) and advanced UC treated with peritoneal dialysis, with LV maximal wall thickness (LVMWT) over 15 mm as determined by CMR [16, 17]. The inclusion criteria of HTN patients were: receiving treatment for hypertension (systolic blood pressure > 140 mmHg or diastolic blood pressure > 90 mmHg) and the presence of concentric LVMWT defined as > 12 mm in the basal septal and inferolateral segments and without evidence of dilated LV cavity on transthoracic echocardiography [18–20]. The inclusion criteria of HCM patients were: adults without HCM family history and LVMWT ≥ 15 mm or adults with HCM family history and LVMWT ≥ 13 mm [21–23]. Healthy controls consisted of normotensive, age and gender-matched healthy subjects, not taking any medications, normal routine blood tests, normal urine samples, and normal CMR findings including normal LV mass indices. The exclusion criteria were: patients with other hypertrophic phenocopies diseases (*n* = 43); significant (≥ grade III) valvular heart disease (*n* = 13); HCM patients with previous septal ablation (*n* = 4); HTN with evidence of dilated LV cavity on transthoracic echocardiography (*n* = 3); degraded CMR image quality (*n* = 5); lack of clinical data (*n* = 9); LV ejection fraction < 50% (*n* = 6) [24] (Fig. 1). The protocol was reviewed and approved by the institution ethics review board of the First Hospital of China Medical University (IRB number: KT2021213). Written informed consent was obtained from all participants.

### CMR image acquisition

All scans were conducted on 3.0-T MRI. Phantom quality assurance tests were conducted every six months to monitor whether parametric mapping values were drifting over time. At the end of expiration, all individuals had a single breath-holding scan with a heart rate ranging from 60 beats/min to 90 beats/min. Short axis stacks were considered a standard method to quantify LV volume and function, T1, T2, and T2* mapping order from base to apex. For T1 quantification, a modified look-locker inversion recovery sequence was implemented, while T2 quantification utilized a T2-prepared balanced steady-state free precession.

### CMR image analysis

**Fig. 2:**
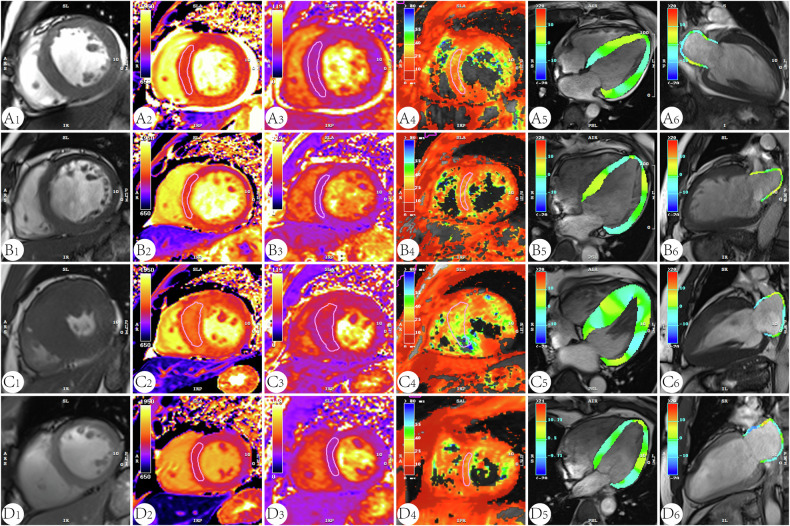
Quantitative cardiac MRI measurements in a UC patient (**A**), an HTN patient (**B**), an HCM patient (**C**), and a healthy subject (**D**). A_1_, B_1_, C_1_, and D_1_ a single short-axis stack; A_2–4_, B_2–4_, C_2–4_, and D_2–4_ quantitative CMR measurements of native T1, T2, and T2* series; A_5_, B_5_, C_5_, and D_5_ 4-chamber view of LA systolic phase; A_6_, B_6_, C_6_, and D_6_ 2-chamber view of LA systolic phase (the LA color varies with different phases and strain parameters)

Functional and parametric analysis of CMR was performed by two radiologists (Z.T. and T.L., with more than 3 years and 15 years of CMR diagnosis experience) blinded using CVI42 software (version 5.3.4, Circle Cardiovascular Imaging, Canada). All individuals were assigned a sequential number and analyzed blindly. LA and LV deformation, including strain and strain rates, were derived via a feature tracking algorithm after the manual delineation of the LA and LV contours, avoiding the inclusion of pulmonary veins. This process facilitated the automatic generation of strain and strain rate variation curves for the cardiac cycle by the CMR-FT module within CVI42 (version 5.3.4, Circle Cardiovascular Imaging, Canada). To ensure precision, all delineated contours were visually examined and adjusted as necessary. All functional and strain parameters for the three groups of patients and healthy controls are shown in Tables 2 and 3. LV and LA epicardial and endocardial borders were automatically traced at both end-diastole and end-systole to calculate cardiac function and myocardial deformation parameters, with manual adjustments as needed. For mapping images (T1, T2, and T2*), short axis stacks were utilized to match regions of interest (ROIs) placed on hypertrophic myocardium, with manual motion correction. HCM ROIs were inserted at the location of local myocardial hypertrophy, whereas UC and HTN ROIs were implanted at the interventricular septum. Figure 2 depicts typical instances from each of the four groups.

**Table 2:** CMR-based cardiac function parameters and myocardial characteristics

Variables	UC, (*n* = 53)	HTN, (*n* = 39)	HCM, (*n* = 30)	Control, (*n* = 30)	*p*
LVEDVI (mL/m^2^)	90.73 ± 24.36^b,c^	102.76 ± 37.24^c^	116.35 ± 37.94^c^	64.67 ± 11.16	0.049
LVESVI (mL/m^2^)	40.49 ± 14.51^c^	47.11 ± 22.17^c^	48.45 ± 26.49^c^	24.34 ± 4.84	< 0.001
LVSVI (mL/m^2^)	50.24 ± 13.13^b,c^	52.36 ± 18.89^c^	63.54 ± 14.68^c^	40.33 ± 8.27	0.025
LVEF (%)	56.03 ± 6.83^c^	53.27 ± 13.54^c^	59.29 ± 14.32	63.49 ± 2.92	0.044
LVMI (g/m^2^)	77.43 ± 20.43^c^	69.96 ± 25.69^c^	73.50 ± 26.49^c^	44.79 ± 7.77	< 0.001
LVMASS (g)	144.19 ± 43.89^c^	127.30 ± 47.67^c^	129.75 ± 47.84^c^	82.72 ± 16.84	< 0.001
HR (bpm)	73.28 ± 9.94	71.08 ± 11.32	67.57 ± 11.9	70.45 ± 12.92	0.057
T1 (ms)	1342.83 ± 49.17^a,b,c^	1295.00 ± 60.53^c^	1285.77 ± 67.10^c^	1189.80 ± 21.43	< 0.001
T2 (ms)	43.79 ± 2.53^a,b,c^	41.51 ± 3.47^c^	41.77 ± 2.21^c^	37.97 ± 1.33	0.002
T2 STAR (ms)	23.12 ± 5.51	22.62 ± 5.47	25.10 ± 6.87	21.29 ± 3.64	0.061

*UC* uremic cardiomyopathy, *HTN* hypertension, *HCM* hypertrophic cardiomyopathy, *LVEDVI* left ventricular end-diastole volume index, *LVESVI* left ventricular end-systolic volume, *LVSVI* left ventricular stroke volume index, *LVEF* left ventricular ejection fraction, *LVMI* left ventricular mass index, *LVMASS* left ventricular mass, *HR* heart rate

^a^ *p* < 0.05 vs HTN

^b^ *p* < 0.05 vs HCM

^c^ *p* < 0.05 vs control

**Table 3:** LV and LA strains and strain rates

Variables	UC, (*n* = 53)	HTN, (*n* = 39)	HCM, (*n* = 30)	Control, (*n* = 30)	*p*
LV-GLS (%)	−11.53 ± 3.04^c^	−12.40 ± 3.62^c^	−10.59 ± 4.33^c^	−17.22 ± 2.18	< 0.001
LV-GCS (%)	−17.90 ± 3.07^a,c^	−15.34 ± 4.58^c^	−15.77 ± 4.10^c^	−19.97 ± 1.96	0.036
LV-GRS (%)	29.83 ± 7.49^c^	25.00 ± 10.59^c^	26.73 ± 9.74^c^	35.49 ± 5.94	0.014
LV-GLSR (s^−^^1^)	−0.79 ± 0.32^b^	−0.74 ± 0.28^c^	−0.62 ± 0.24^c^	−0.92 ± 0.16	0.008
LV-GCSR (s^−^^1^)	−0.93 ± 0.19	−0.89 ± 0.37^c^	−0.95 ± 0.38	−1.05 ± 0.20	0.002
LV-GRSR (s^−^^1^)	1.61 ± 0.49	1.42 ± 0.72^c^	1.87 ± 1.23	1.89 ± 0.42	< 0.001
LA-RS (%)	29.08 ± 13.00^c^	22.83 ± 10.76^c^	22.31 ± 12.48^c^	42.59 ± 11.63	< 0.001
LA-CS (%)	15.97 ± 14.10	14.69 ± 7.52^c^	12.64 ± 7.11^c^	20.79 ± 8.92	0.007
LA-BS (%)	13.36 ± 10.61^c^	10.45 ± 5.52^c^	10.96 ± 5.68^c^	20.40 ± 7.67	< 0.001
LA-RSR (s^−^^1^)	1.64 ± 0.72^a,b,c^	1.22 ± 0.63^c^	1.04 ± 0.61^c^	2.28 ± 0.57	< 0.001
LA-CSR (s^−^^1^)	−1.68 ± 1.13^a,b,c^	−0.98 ± 0.53^c^	−1.01 ± 0.71^c^	−2.78 ± 1.29	< 0.001
LA-BSR (s^−^^1^)	−1.92 ± 1.01	−1.56 ± 0.92^c^	−1.37 ± 1.01^c^	−2.33 ± 0.75	< 0.001

*UC* uremic cardiomyopathy, *HTN* hypertension, *HCM* hypertrophic cardiomyopathy, *LV-GLS* left ventricular global longitudinal strain, *LV-GCS* left ventricular global circumferential strain, *LV-GRS* left ventricular global radial strain, *LV-GLSR* left ventricular global longitudinal strain rate, *LV-GCSR* left ventricular global circumferential strain rate, *LV-GRSR* left ventricular global radial strain rate, *LA-RS* left atrial reservoir strain, *LA-CS* left atrial conduit strain, *LA-BS* left atrial booster strain, *LA-RSR* left atrial reservoir strain rate, *LA-CSR* left atrial conduit strain rate, *LA-BSR* left atrial booster strain rate

^a^ *p* < 0.05 vs HTN

^b^ *p* < 0.05 vs HCM

^c^ *p* < 0.05 vs control

### Statistical analysis

Quantitative data were presented as mean ± standard deviation (SD), and median (interquartile range), while categorical data were expressed as counts with corresponding percentages. Tests for normality and variance homogeneity were applied to the quantitative variables. For normally distributed quantitative variables, comparisons were conducted using the ANOVA test, whereas the Kruskal–Wallis rank-sum test was employed for non-normally distributed variables, while categorical variables were compared using the Chi-square or Fisher’s exact test. The effects of clinical parameters and CMR parameters were evaluated using covariance analysis. The relationship between quantitative variables and primary CMR findings was assessed using Pearson or Spearman rank correlation methods. The corrections between clinical outcomes and CMR characteristics were assessed using univariate and multivariate logistic regression. Statistical significance was determined using a two-tailed test with a *p* value < 0.05.

## Results

### Baseline characteristics and anthropometric variables

The baseline characteristics and anthropometric variables are summarized in Table 1. A total of 152 subjects who underwent CMR were included in this study, including 53 UC patients (mean age, 46.4 ± 8.7 years; 78.4% male), 39 HTN patients (mean age, 51.9 ± 11.0 years; 57.9% male), 30 HCM patients (mean age, 51.3 ± 9.4 years; 60.0% male), and 30 healthy controls (mean age, 48.3 ± 10.0 years; 63.3% male). The flowchart of the study population is shown in Fig. 1. There was no significant difference in age, sex, body mass index (BMI), high-sensitivity cardiac troponin I (hsTnI), dyslipidemia, diabetes mellitus, and smoking history among the four groups. UC and HTN patients had a lower brain natriuretic peptide (BNP) than HCM patients (148.00 [84.00–287.00] vs 537.50 [245.50–870.50] pg/mL; 101.00 [43.00–187.00] vs 537.50 [245.50–870.50], *p* < 0.001; *p* = 0.002). Systolic and diastolic blood pressures were significantly elevated in the UC and HTN groups compared to the HCM and control groups. Specifically, UC and HTN patients had higher systolic and diastolic blood pressures (146.62 ± 12.81 mmHg and 95.28 ± 10.90 mmHg; 140.59 ± 11.19 mmHg, and 90.15 ± 5.29 mmHg) compared to HCM (128.67 ± 8.65 mmHg and 75.77 ± 6.80 mmHg) and control groups (111.5 ± 10.02 mmHg and 75.13 ± 7.24 mmHg) (*p* < 0.001 and *p* = 0.023, respectively).

### CMR-based myocardial characteristics

**Fig. 3:**
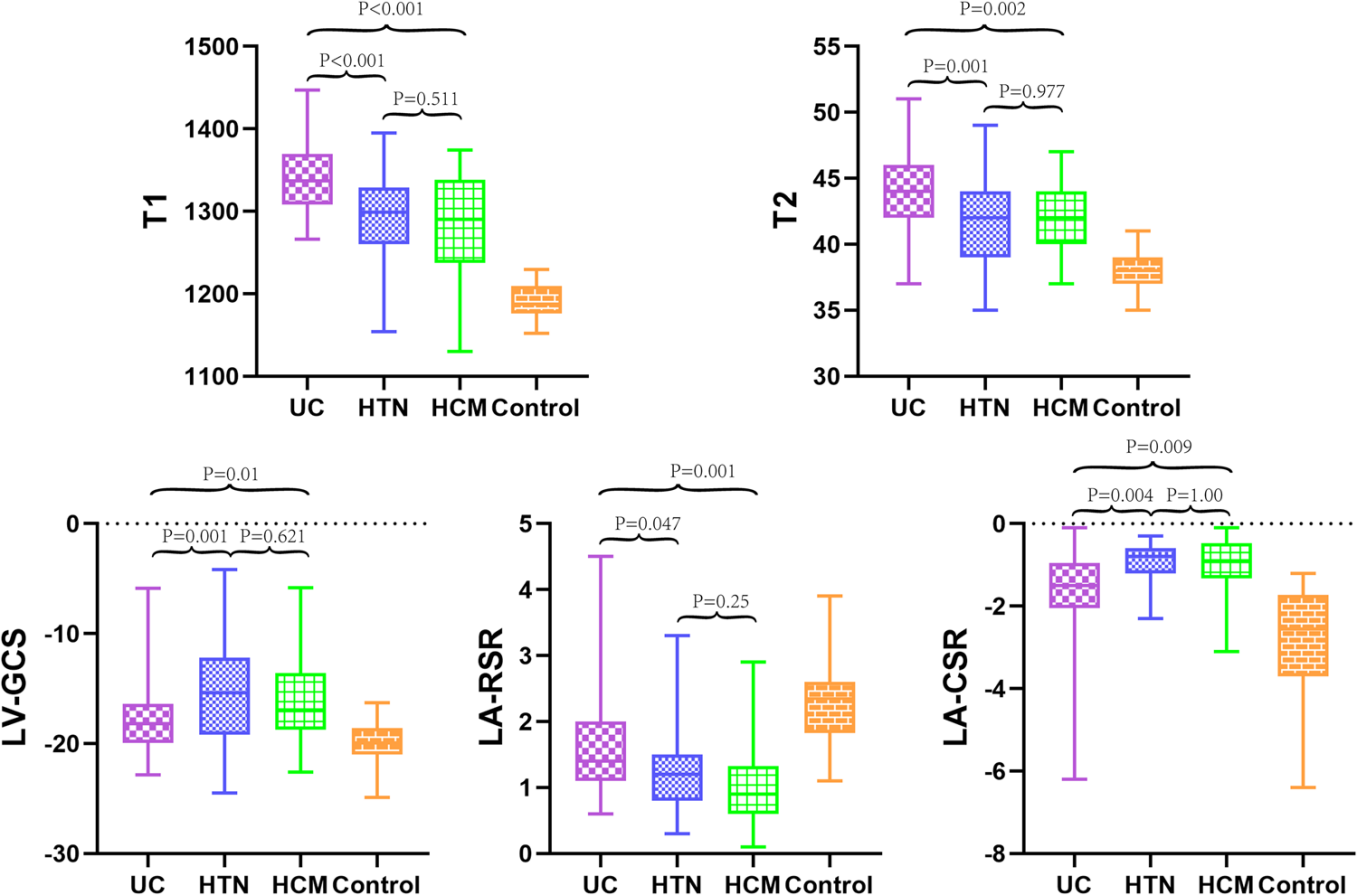
Group-specific mean values of native T1 and T2 in the study groups. UC, uremic cardiomyopathy; HTN, hypertension; HCM, hypertrophic cardiomyopathy; LV-GCS, left ventricular global radial strain; LA-RSR, left atrial reservoir strain rate; LA-CSR, left atrial conduit strain rate

The myocardial characteristics derived from CMR for the three patient groups and healthy controls are presented in Table 2. The three groups of patients had higher left ventricular end-diastole volume index (LVEDVI), left ventricular end-systolic volume (LVESVI), left ventricular stroke volume index (LVSVI), left ventricular mass index (LVMI) and left ventricular mass (LVMASS) than healthy controls (*p* < 0.01 for all). Whereas the UC patients’ LVEDVI and LVSVI were lower than HCM patients (*p* < 0.05). There was no significant difference in HR between the four groups, as well as in LVESVI, LVEF, and LVMI between the UC, HTN, and HCM patients. Native T1 and T2 values were increased in UC, HTN, and HCM patients compared with healthy controls (*p* < 0.01 for all). UC patients had significantly higher native T1 (1342.83 ± 49.17 vs 1295.00 ± 60.53; 1342.83 ± 49.17 vs 1285.77 ± 67.10; 1342.83 ± 49.17 vs 1189.80 ± 21.43, *p* < 0.001 for all) and T2 (43.79 ± 2.53 vs 41.51 ± 3.47; 43.79 ± 2.53 vs 41.77 ± 2.21; 43.79 ± 2.53 vs 37.97 ± 1.33, *p* < 0.002 for all) values compared with HTN and HCM patients (Fig. 3). The covariance analysis revealed that, after adjusting for baseline systolic and diastolic blood pressures, significant differences in myocardial T1 and T2 values persist among the different disease groups (Supplementary Tables 1–4). There was no significant difference in native T2* values between the four groups.

### LV and LA strains and strain rates

LV and LA strains and strain rates derived by CMR-FT are presented in Table 3. The absolute values of left ventricular global longitudinal strain (LV-GLS), left ventricular global circumferential strain (LV-GCS), and left ventricular global radial strain (LV-GRS) were lower in patient groups as compared with healthy controls (*p* < 0.05 for all). The absolute value of LV-GCS in UC patients was higher than that in HTN patients (−17.90 ± 3.07 vs −15.34 ± 4.58, *p* < 0.05). The absolute value of LV-GLS rate (LV-GLSR) in UC patients was higher than that in HCM patients (−0.79 ± 0.32 vs −0.62 ± 0.24, *p* < 0.01). The absolute values of LV-GCS rate (LV-GCSR) and LV-GRS rate (LV-GRSR) in HTN patients were lower than that in healthy controls (*p* < 0.01 for all). All absolute values of LA reservoir strain (LA-RS), conduit strain (LA-CS), booster strain (LA-BS), and their strain rates were lower in patient groups as compared with those values in the healthy control subject, except for LA-CS and LA-BS rate (LA-BSR) of UC patients (*p* < 0.05 for all). The absolute values of LA-RS rate (LA-RSR) (1.64 ± 0.72 vs 1.22 ± 0.63; 1.64 ± 0.72 vs 1.04 ± 0.61, *p* < 0.001 for all) and LA-CS rate (LA-CSR) (−1.68 ± 1.13 vs −0.98 ± 0.53; −1.68 ± 1.13 vs −1.01 ± 0.71, *p* < 0.001 for all) were higher in UC patients than in HTN and HCM patients.

### Analysis of relationships

**Fig. 4:**
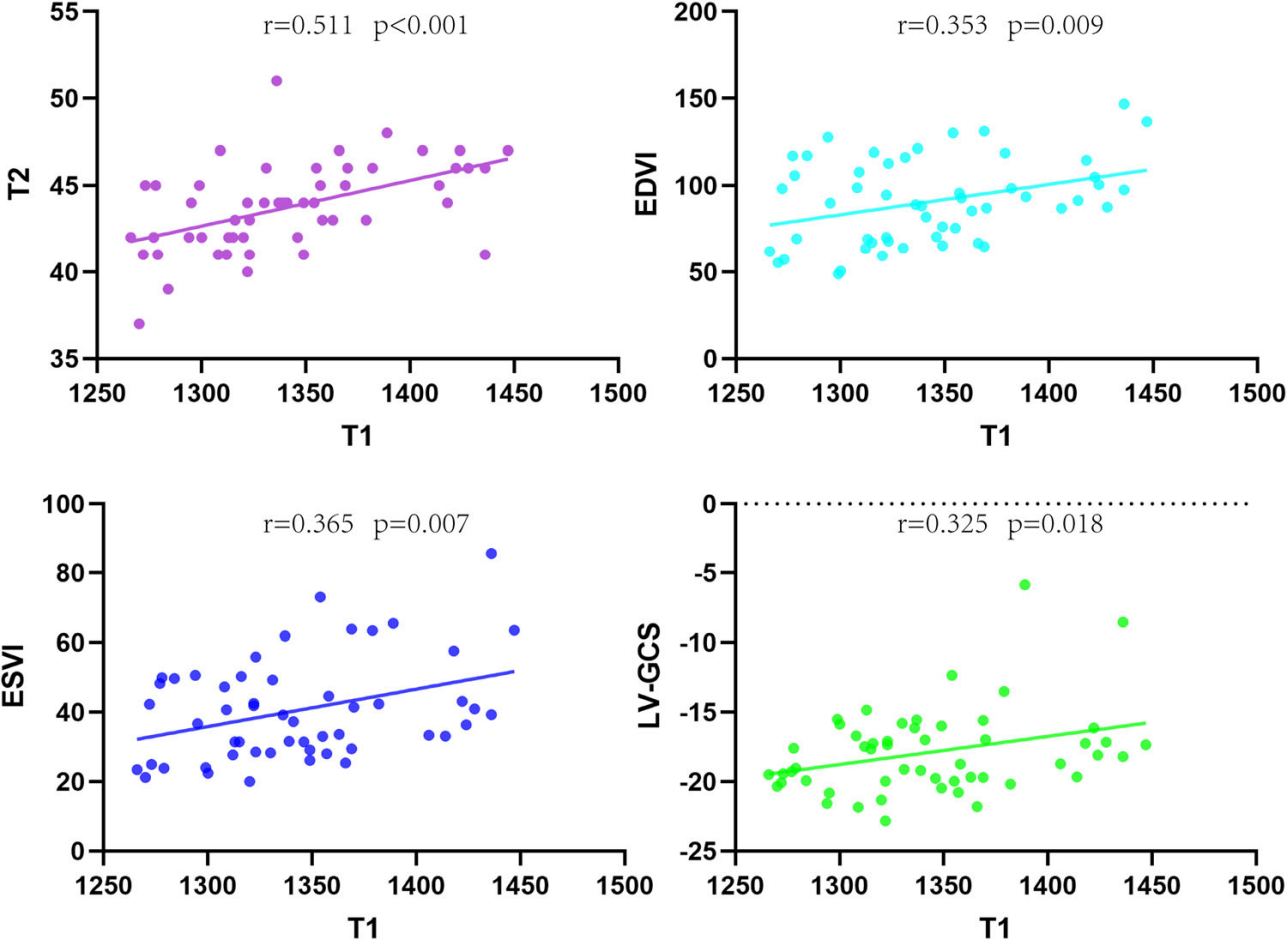
Association between native T1 and T2, EDVI, ESVI, and LV-GCS. Specific *r* values are included. EDVI, end-diastole volume index; ESVI, end-systolic volume; LV-GCS, left ventricular global circumferential strain

According to the Pearson correlation coefficient, there was a moderate correlation between T1 and T2 values in UC patients (*r* = 0.511, *p* < 0.001) while no such correlation between T1 and T2 values was found in the other three groups. There were weak correlations between T1 and EDVI values, T1 and ESVI values in UC patients (*r* = 0.353, *p* < 0.01; *r* = 0.365, *p* < 0.01) while no statistical correlations between those values were found in the other three groups. There were weak correlations between T1 and GCS in UC and HTN patients (*r* = 0.325, *p* = 0.018; *r* = 0.319, *p* = 0.048) while no statistical correlations between those parameters were found in the other two groups (Fig. 4).

### ROC analysis of LV and LA parameters for differentiating UC patients from HTN and HCM patients

**Fig. 5:**
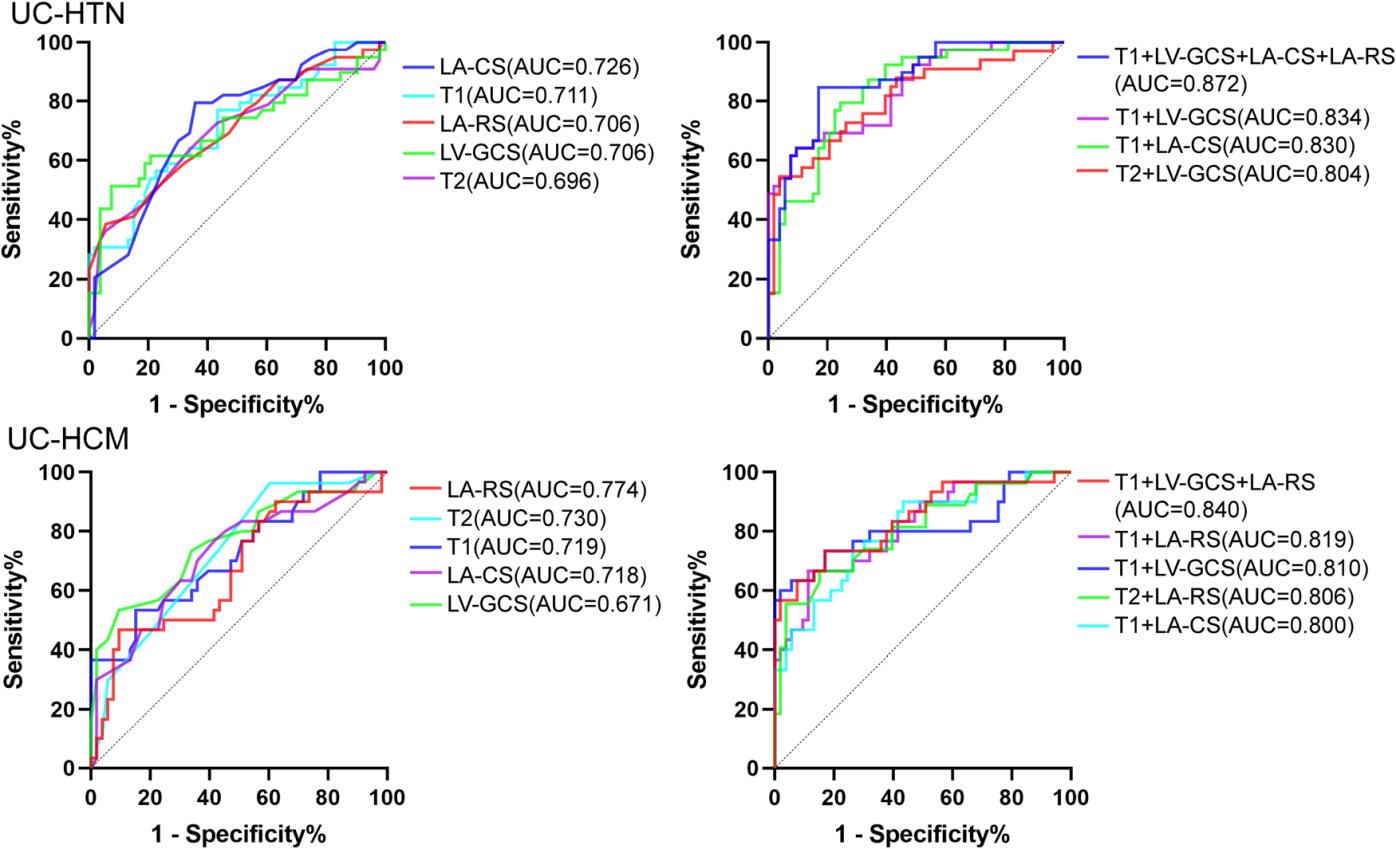
ROC curve analysis of LV and LA parameters for differentiating UC patients from the other two groups. UC, uremic cardiomyopathy; HTN, hypertension; HCM, hypertrophic cardiomyopathy; LA-CS, left atrial conduit strain; LA-RS, left atrial reservoir strain; LV-GCS, left ventricular global circumferential strain

T1, LA-RSR and LV-GCS (area under ROC curve [AUC] 0.711, 95% confidence interval [CI]: 0.604–0.819; AUC 0.706, 95% CI: 0.597–0.815; AUC 0.706, 95% CI: 0.591–0.821; respectively, *p* < 0.01 for all) were distinguishing between UC and HTN patients (Fig. 5). T1, T2, LA-RSR and LA-CSR (AUC 0.719, 95% CI: 0.598–0.841; AUC 0.730, 95% CI: 0.617–0.843; AUC 0.774, 95% CI: 0.663–0.885; AUC 0.718, 95% CI: 0.600–0.836; respectively, *p* < 0.01 for all) were distinguishing between UC and HCM patients. The combination of T1, LV-GCS, LA-CS, and LA-RS (AUC 0.872, 95% CI: 0.801–0.943) was the best distinguishing feature between UC and HTN patients. The combination of T1, LV-GCS, and LA-RS was the best distinguishing feature between UC and HCM patients (AUC 0.840, 95% CI: 0.746–0.934).

## Discussion

This study compared myocardial tissue features and strain parameters of the left ventricle and left atrium in patients with UC, HTN, and HCM in comparison with healthy control subjects. In previous studies, researchers have usually focused on patients’ LV function and native T1 and T2 values, but rarely paid attention to LA function and T2* values [11, 25]. We have incorporated all the above indicators into our study. The main findings of our study are: (1) native T1 and T2 values are higher in patients with UC as compared to those values in patients with HTN and HCM. And there was also a moderate correlation between them. (2) LA-RS and LA-CS values were higher in patients with UC as compared to the other two hypertrophic phenotypes. (3) The combination of myocardial T1 values and strain parameters was a distinguishing feature between UC and the other two hypertrophic phenotypes.

We found that myocardial T1 and T2 values are higher in UC. In addition, we found a moderate correlation between native T1 and T2 values in UC. In contrast to UC, no such correlation was found in the other two hypertrophic phenotypes. The combination of native T1 values and extracellular volume fraction can reflect myocardial fibrosis. Correlated native T1 and T2 values reflect myocardial edema [26, 27]. Daniel R. Messroghli and colleagues determined that T1 and T2 mapping are equivalently efficacious only in the detection of edema [7]. Thus, edema is more pronounced in UC patients as compared to HTN and HCM patients. Volume overload may play an additional role in enhancing myocardial edema in patients with UC. It has been shown that myocardial T1 and T2 values vary dynamically with humoral volume change in patients on hemodialysis [28]. However, all the patients in our study were treated with peritoneal dialysis to make sure that their humoral volume change was relatively stable. Furthermore, in our study myocardial T2* values as a measure of iron deposition were within the normal range in UC, as well as the other two hypertrophic phenotypes.

Strain and strain rate represents the magnitude and rate of myocardial deformation and may be early markers of cardiac dysfunction. In our study, the absolute value of LV-GCS was significantly higher in UC patients as compared to those values in hypertensive patients. The absolute value of GLSR was significantly higher in UC patients as compared to that measurement in patients with HCM. Apparently, LV function is better preserved in UC as compared to the other two cardiomyopathies. Furthermore, LA strain rates showed in our study significant differences between UC patients and the other two patient groups. LA dysfunction is an early marker of LV dysfunction and prognosis in various cardiac diseases [29, 30]. The LA works as a reservoir for pulmonary venous return during ventricular systole, a conduit for pulmonary venous return during early ventricular diastole, and a booster pump to increase ventricular filling during late ventricular diastole [31]. Reservoir function is mainly affected by atrial compliance during ventricular systole, it is influenced by the decrease of LV base during systole, as well as LV end-systolic volume [32]. Conduit function is affected by atrial compliance, LV relaxation, and compliance. Finally, the atrial booster pump function reflects the magnitude and timing of atrial contractility and depends on venous reflux, LV end-diastolic pressures, and LV systolic reserve [31]. LA strain has high-sensitivity in identifying raised atrial stiffness and wall fibrosis [33, 34]. It plays an important role in the evaluation of LA function. In our study, we mainly estimated reservoir strain, conduit strain, and booster strain to measure the corresponding function. LA-RSR and LA-CSR were significantly higher in UC patients as compared to those values in HTN and HCM patients. Our findings suggest that LA reservoir and conduit function are better preserved in UC patients as compared to HTN and HCM patients. Apparently, UC patients have better preserved LA compliance, LV compliance, and LV relaxation.

We analyzed myocardial and strain parameters that may be helpful in distinguishing UC from hypertensive and HCM. The combined analysis of T1, LV-GCS, LA-CS, and LA-RS was the best-performing distinguishing feature between uremic and HTN. Furthermore, the combined analysis of T1, LV-GCS, and LA-RS was best-performing to distinguish uremic and HCM.

Finally, our findings suggest potential improvements in diagnosing and managing UC. The distinct myocardial T1, T2 and strain parameters in UC patients compared to other cardiomyopathies could lead to more accurate diagnostic criteria enabling earlier identification and timely intervention. Additionally, the potential reversibility of myocardial changes associated with edema in UC warrants further investigation, potentially leading to therapies targeting edema reduction to improve cardiac function. The noninvasive nature of CMR allows for regular monitoring and personalized treatment adjustments. While myocardial biopsy is not routinely recommended for UC, HTN or HCM, future studies could validate our findings by correlating CMR data with myocardial biopsy results in a subset of patients where biopsy is feasible and clinically indicated [35–39]. Furthermore, a longitudinal follow-up of these patients could provide valuable insights into the progression of UC and its comparison with HTN and HCM over time. This approach could help understand the disease trajectory and response to treatments, further enhancing clinical decision-making. These findings underscore CMR’s value in clinical decision-making and highlight the need for further prospective studies to validate and integrate these results into routine practice.

### Limitations

This is a retrospective single-center study with a relatively small sample size, we need prospective studies in large and broad populations to validate our findings for exploring the clinical utility of patient management. Potential biases, such as selection bias, might have influenced the results since our study population may not represent the broader patient population. Additionally, the retrospective nature of the study introduces the risk of information bias due to variability in data quality and completeness, which could result in inaccuracies or inconsistencies. Owing to the fact that myocardial biopsy is not conducted as a routine procedure, we were unable to evaluate the correlation between myocardial histology and CMR parameters. In addition, UC patients can not accept enhanced scans considering their safety so we did not evaluate the myocardial extracellular volume and late gadolinium. Still, we can get multiple parameters in a noninvasive way without contrast.

## Conclusions

We explored the presence and extent of CMR tissue characteristics and cardiac strain in UC in contrast to hypertensive and HCM. We found distinguishing differences in myocardial T1 and T2 values and strain parameters in UC patients as compared to the other two cardiomyopathies. Myocardial seems to be more apparent in UC as compared to the other two cardiomyopathies. Strain analysis revealed better preserved LA and ventricular function as compared to the other two patient groups. Finally, we explored distinguishing features based on the myocardial tissue characteristics and strain between UC and hypertensive and HCM. The combined analysis of myocardial T1 values and strain revealed promise to distinguish UC from the two other phenotypes. Our findings suggest that the observed differences in myocardial tissue characteristics between UC, HTN, and HCM patients may be indicative of underlying etiological distinctions. However, these hypotheses require further investigation to establish causality and to understand the full clinical implications.

### Supplementary information

ELECTRONIC SUPPLEMENTARY MATERIAL

## Data Availability

The datasets used or analyzed during the current study are available from the corresponding author upon reasonable request.

## Abbreviations

AUC: Area under ROC curve
BMI: Body mass index
BNP: Brain natriuretic peptide
CMR: Cardiac magnetic resonance imaging
CMR-FT: CMR feature tracking
CI: Confidence interval
ESRD: End-stage renal disease
hsTnI: High-sensitivity cardiac troponin I
HTN: Hypertensive cardiomyopathy
HCM: Hypertrophic cardiomyopathy
LA-CS: LA conduit strain
LA-RS: LA reservoir strain
LA-BSR: LA-BS rate
LA-CSR: LA-CS rate
LA-RSR: LA-RS rate
LA: Left atrial
LV: Left ventricular
LVEDVI: Left ventricular end-diastole volume index
LVESVI: Left ventricular end-systolic volume
LV-GCS: Left ventricular global circumferential strain
LV-GLS: Left ventricular global longitudinal strain
LV-GRS: Left ventricular global radial strain
LVMASS: Left ventricular mass
LVMI: Left ventricular mass index
LVMWT: Left ventricular maximal wall thickness
LVSVI: Left ventricular stroke volume index
LV-GCSR: LV-GCS rate
LV-GLSR: LV-GLS rate
LV-GRSR: LV-GRS rate
ROI: Regions of interest
UC: Uremic cardiomyopathy

## References

[1] Luyckx VA, Tonelli M, Stanifer JW (2018) The global burden of kidney disease and the sustainable development goals. Bull World Health Organ 96:414–422D 29904224 10.2471/BLT.17.206441 PMC5996218

[2] GBD 2015 Mortality and Causes of Death Collaborators (2016) Global, regional, and national life expectancy, all-cause mortality, and cause-specific mortality for 249 causes of death, 1980-2015: a systematic analysis for the Global Burden of Disease Study 2015. Lancet 388:1459–1544 27733281 10.1016/S0140-6736(16)31012-1 PMC5388903

[3] Shamseddin MK, Parfrey PS (2011) Sudden cardiac death in chronic kidney disease: epidemiology and prevention. Nat Rev Nephrol 7:145–154 21283136 10.1038/nrneph.2010.191

[4] Ma D, Mandour AS, Elfadadny A et al (2022) Changes in cardiac function during the development of uremic cardiomyopathy and the effect of salvianolic acid B administration in a rat model. Front Vet Sci 9:905759 35782566 10.3389/fvets.2022.905759 PMC9244798

[5] Valbuena-López SC, Camastra G, Cacciotti L et al (2023) Cardiac imaging biomarkers in chronic kidney disease. Biomolecules 13:773 37238643 10.3390/biom13050773 PMC10216582

[6] Taylor AJ, Salerno M, Dharmakumar R, Jerosch-Herold M (2016) T1 mapping: basic techniques and clinical applications. JACC Cardiovasc Imaging 9:67–81 26762877 10.1016/j.jcmg.2015.11.005

[7] Messroghli DR, Moon JC, Ferreira VM et al (2017) Clinical recommendations for cardiovascular magnetic resonance mapping of T1, T2, T2* and extracellular volume: a consensus statement by the Society for Cardiovascular Magnetic Resonance (SCMR) endorsed by the European Association for Cardiovascular Imaging (EACVI). J Cardiovasc Magn Reson 19:75 28992817 10.1186/s12968-017-0389-8 PMC5633041

[8] Carpenter J-P, He T, Kirk P et al (2011) On T2* magnetic resonance and cardiac iron. Circulation 123:1519–1528 21444881 10.1161/CIRCULATIONAHA.110.007641 PMC3435874

[9] Kali A, Kumar A, Cokic I et al (2013) Chronic manifestation of postreperfusion intramyocardial hemorrhage as regional iron deposition: a cardiovascular magnetic resonance study with ex vivo validation. Circ Cardiovasc Imaging 6:218–228 23403335 10.1161/CIRCIMAGING.112.000133

[10] Xu J, Yang W, Zhao S, Lu M (2022) State-of-the-art myocardial strain by CMR feature tracking: clinical applications and future perspectives. Eur Radiol 32:5424–5435 35201410 10.1007/s00330-022-08629-2

[11] Cavus E, Muellerleile K, Schellert S et al (2021) CMR feature tracking strain patterns and their association with circulating cardiac biomarkers in patients with hypertrophic cardiomyopathy. Clin Res Cardiol 110:1757–1769 33779809 10.1007/s00392-021-01848-5 PMC8563550

[12] Neisius U, Myerson L, Fahmy AS et al (2019) Cardiovascular magnetic resonance feature tracking strain analysis for discrimination between hypertensive heart disease and hypertrophic cardiomyopathy. PLoS One 14:e0221061 31433823 10.1371/journal.pone.0221061 PMC6703851

[13] Nazir SA, Shetye AM, Khan JN et al (2020) Inter-study repeatability of circumferential strain and diastolic strain rate by CMR tagging, feature tracking and tissue tracking in ST-segment elevation myocardial infarction. Int J Cardiovasc Imaging 36:1133–1146 32152811 10.1007/s10554-020-01806-8 PMC7228913

[14] Sucharov CC (2020) Paracrine factors in uremic cardiomyopathy. JACC Basic Transl Sci 5:167–168 32142069 10.1016/j.jacbts.2020.01.005 PMC7046544

[15] Kaesler N, Babler A, Floege J, Kramann R (2020) Cardiac remodeling in chronic kidney disease. Toxins 12:161 32150864 10.3390/toxins12030161 PMC7150902

[16] Kawel-Boehm N, Hetzel SJ, Ambale-Venkatesh B et al (2020) Reference ranges (“normal values”) for cardiovascular magnetic resonance (CMR) in adults and children: 2020 update. J Cardiovasc Magn Reson 22:87 33308262 10.1186/s12968-020-00683-3 PMC7734766

[17] Chen TK, Knicely DH, Grams ME (2019) Chronic kidney disease diagnosis and management: a review. JAMA 322:1294–1304 31573641 10.1001/jama.2019.14745 PMC7015670

[18] Mancia G, Fagard R, Narkiewicz K et al (2013) 2013 ESH/ESC guidelines for the management of arterial hypertension: the task force for the management of arterial hypertension of the European Society of Hypertension (ESH) and of the European Society of Cardiology (ESC). J Hypertens 31:1281–1357 23817082 10.1097/01.hjh.0000431740.32696.cc

[19] Whelton PK, Carey RM, Aronow WS et al (2018) 2017 ACC/AHA/AAPA/ABC/ACPM/AGS/APhA/ASH/ASPC/NMA/PCNA guideline for the prevention, detection, evaluation, and management of high blood pressure in adults: a report of the American College of Cardiology/American Heart Association Task Force on Clinical Practice Guidelines. J Am Coll Cardiol 71:e127–e248 29146535 10.1016/j.jacc.2017.11.006

[20] Joint Committee for Guideline Revision (2019) 2018 Chinese guidelines for prevention and treatment of hypertension—a report of the revision committee of Chinese guidelines for prevention and treatment of hypertension. J Geriatr Cardiol 16:182–241 31080465 10.11909/j.issn.1671-5411.2019.03.014 PMC6500570

[21] Makavos G, Κairis C, Tselegkidi M-E et al (2019) Hypertrophic cardiomyopathy: an updated review on diagnosis, prognosis, and treatment. Heart Fail Rev 24:439–459 30852773 10.1007/s10741-019-09775-4

[22] Baessato F, Romeo C, Rabbat MG et al (2022) A comprehensive assessment of cardiomyopathies through cardiovascular magnetic resonance: focus on the pediatric population. Diagnostics 12:1022 35626178 10.3390/diagnostics12051022 PMC9139185

[23] Authors/Task Force members, Elliott PM, Anastasakis A et al (2014) 2014 ESC guidelines on diagnosis and management of hypertrophic cardiomyopathy: the task force for the diagnosis and management of hypertrophic cardiomyopathy of the European Society of Cardiology (ESC). Eur Heart J 35:2733–2779 25173338 10.1093/eurheartj/ehu284

[24] Li H, Zheng Y, Peng X et al (2023) Heart failure with preserved ejection fraction in post myocardial infarction patients: a myocardial magnetic resonance (MR) tissue tracking study. Quant Imaging Med Surg 13:1723–1739 36915319 10.21037/qims-22-793 PMC10006144

[25] Patel AR, Kramer CM (2017) Role of cardiac magnetic resonance in the diagnosis and prognosis of nonischemic cardiomyopathy. JACC Cardiovasc Imaging 10:1180–1193 28982571 10.1016/j.jcmg.2017.08.005 PMC5708889

[26] Arcari L, Hinojar R, Engel J et al (2020) Native T1 and T2 provide distinctive signatures in hypertrophic cardiac conditions—comparison of uremic, hypertensive and hypertrophic cardiomyopathy. Int J Cardiol 306:102–108 32169347 10.1016/j.ijcard.2020.03.002

[27] Pradella S, Grazzini G, De Amicis C et al (2020) Cardiac magnetic resonance in hypertrophic and dilated cardiomyopathies. Radiol Med 125:1056–1071 32946001 10.1007/s11547-020-01276-x

[28] Kotecha T, Martinez-Naharro A, Yoowannakul S et al (2019) Acute changes in cardiac structural and tissue characterisation parameters following haemodialysis measured using cardiovascular magnetic resonance. Sci Rep 9:1388 30718606 10.1038/s41598-018-37845-4 PMC6362126

[29] Thomas L, Marwick TH, Popescu BA et al (2019) Left atrial structure and function, and left ventricular diastolic dysfunction: JACC state-of-the-art review. J Am Coll Cardiol 73:1961–1977 31000000 10.1016/j.jacc.2019.01.059

[30] Frydas A, Morris DA, Belyavskiy E et al (2020) Left atrial strain as sensitive marker of left ventricular diastolic dysfunction in heart failure. ESC Heart Fail 7:1956–1965 32613770 10.1002/ehf2.12820 PMC7373910

[31] Hoit BD (2014) Left atrial size and function: role in prognosis. J Am Coll Cardiol 63:493–505 24291276 10.1016/j.jacc.2013.10.055

[32] Barbier P, Solomon SB, Schiller NB, Glantz SA (1999) Left atrial relaxation and left ventricular systolic function determine left atrial reservoir function. Circulation 100:427–436 10421605 10.1161/01.CIR.100.4.427

[33] Cameli M, Mandoli GE, Loiacono F et al (2016) Left atrial strain: a useful index in atrial fibrillation. Int J Cardiol 220:208–213 27389443 10.1016/j.ijcard.2016.06.197

[34] Raafs AG, Vos JL, Henkens MTHM et al (2022) Left atrial strain has superior prognostic value to ventricular function and delayed-enhancement in dilated cardiomyopathy. JACC Cardiovasc Imaging 15:1015–1026 35680209 10.1016/j.jcmg.2022.01.016

[35] Arbelo E, Protonotarios A, Gimeno JR et al (2023) 2023 ESC guidelines for the management of cardiomyopathies. Eur Heart J 44:3503–3626 37622657 10.1093/eurheartj/ehad194

[36] Whelton PK, Carey RM, Aronow WS et al (2018) 2017 ACC/AHA/AAPA/ABC/ACPM/AGS/APhA/ASH/ASPC/NMA/PCNA guideline for the prevention, detection, evaluation, and management of high blood pressure in adults: a report of the American College of Cardiology/American Heart Association Task Force on Clinical Practice Guidelines. Hypertension 71:e13–e115 29133356 10.1161/HYP.0000000000000065

[37] Alhaj E, Alhaj N, Rahman I, Niazi TO, Berkowitz R, Klapholz M (2013) Uremic cardiomyopathy: an underdiagnosed disease. Congest Heart Fail 19:E40–E45 23615021 10.1111/chf.12030

[38] Mark PB, Mangion K, Rankin AJ et al (2022) Left ventricular dysfunction with preserved ejection fraction: the most common left ventricular disorder in chronic kidney disease patients. Clin Kidney J 15:2186–2199 36381379 10.1093/ckj/sfac146 PMC9664574

[39] Ommen SR, Mital S, Burke MA et al (2020) 2020 AHA/ACC guideline for the diagnosis and treatment of patients with hypertrophic cardiomyopathy: executive summary: a report of the American College of Cardiology/American Heart Association Joint Committee on Clinical Practice Guidelines. Circulation 142:e533–e557 33215938 10.1161/CIR.0000000000000938

