# Supplementary material for: Myocardial hypertrophy: the differentiation of uremic, hypertensive, and hypertrophic cardiomyopathies by cardiac MRI
Source: Insights Imaging. 2024 Aug 1;15:190. doi: 10.1186/s13244-024-01770-0 (PMC11294291; doi:10.1186/s13244-024-01770-0)
Supplement: Supplementary file 1 — ELECTRONIC SUPPLEMENTARY MATERIAL [file 13244_2024_1770_MOESM1_ESM.pdf]

Myocardial Hypertrophy: The Differentiation of Uremic, Hypertensive, and Hypertrophic Cardiomyopathies by Cardiac MRI

ELECTRONIC SUPPLEMENTARY MATERIAL

Table of Contents

Supplementary Table 1.Comparison of Group T1 Means and Adjusted Mean Differences with Baseline Systolic Blood Pressure Adjustment 2
Supplementary Table 2.Comparison of Group T1 Means and Adjusted Mean Differences with Baseline Diastolic Blood Pressure Adjustment 3
Supplementary Table 3.Comparison of Group T2 Means and Adjusted Mean Differences with Baseline Systolic Blood Pressure Adjustment 4
Supplementary Table 4.Comparison of Group T2 Means and Adjusted Mean Differences with Baseline Diastolic Blood Pressure Adjustment 5

Supplementary Table 1. Comparison of Group T1 Means and Adjusted Mean Differences with Baseline Systolic Blood Pressure Adjustment

Group	Mean ± Standard Deviation(msec)	Adjusted Mean Difference and 95% Confidence Interval(msec)*	t-test	
			t-value	p-value
UC	1342.83±49.17	130.45(94.81-166.10)	7.23	<0.001
HTN	1295.00±60.53	86.50(53.06-119.95)	5.11	<0.001
HCM	1285.77±67.10	84.74(55.26-114.62)	5.66	<0.001
Control	1189.80±21.43	0[a]	-	-

*Adjusted for baseline systolic blood pressure.

[a]This parameter is redundant and is set to zero.

UC = uremic cardiomyopathy; HTN = hypertension; HCM = hypertrophic cardiomyopathy.

Supplementary Table 2.Comparison of Group T1 Means and Adjusted Mean Differences with Baseline Diastolic Blood Pressure Adjustment

Group	Mean ± Standard Deviation(msec)	Adjusted Mean Difference and 95% Confidence Interval(msec)*	t-test	
			t-value	p-value
UC	1342.83±49.17	133.00(101.71-164.29)	8.40	<0.001
HTN	1295.00±60.53	90.27(60.92-119.63)	6.08	<0.001
HCM	1285.77±67.10	95.34(68.75-121.92)	7.09	<0.001
Control	1189.80±21.43	0[a]	-	-

*Adjusted for baseline diastolic blood pressure.

[a]This parameter is redundant and is set to zero.

UC = uremic cardiomyopathy; HTN = hypertension; HCM = hypertrophic cardiomyopathy.

Supplementary Table 3.Comparison of Group T2 Means and Adjusted Mean Differences with Baseline Systolic Blood Pressure Adjustment

Group	Mean ± Standard Deviation(msec)	Adjusted Mean Difference and 95% Confidence Interval(msec)*	t-test	
			t-value	p-value
UC	43.79±2.53	5.68(3.92-7.45)	6.37	<0.001
HTN	41.51±3.47	3.43(1.77-5.08)	4.09	<0.001
HCM	41.77±2.21	3.73(2.26-5.20)	5.02	<0.001
Control	37.97±1.33	0[a]	-	-

*Adjusted for baseline systolic blood pressure.

[a]This parameter is redundant and is set to zero.

UC = uremic cardiomyopathy; HTN = hypertension; HCM = hypertrophic cardiomyopathy.

Supplementary Table 4.Comparison of Group T2 Means and Adjusted Mean Differences with Baseline Diastolic Blood Pressure Adjustment

Group	Mean ± Standard Deviation(msec)	Adjusted Mean Difference and 95% Confidence Interval(msec)*	t-test	
			t-value	p-value
UC	43.79±2.53	5.88(4.32-7.43)	7.47	<0.001
HTN	41.51±3.47	3.58(2.13-5.04)	4.86	<0.001
HCM	41.77±2.21	3.80(2.48-5.12)	5.69	<0.001
Control	37.97±1.33	0[a]	-	-

*Adjusted for baseline diastolic blood pressure.

[a]This parameter is redundant and is set to zero.

UC = uremic cardiomyopathy; HTN = hypertension; HCM = hypertrophic cardiomyopathy.
